# The safety and efficacy of non-emergent percutaneous coronary intervention via distal radial artery access in patients with acute coronary syndrome

**DOI:** 10.3389/fcvm.2025.1696742

**Published:** 2025-12-19

**Authors:** Dan Niu, Qinggele Gao, Baojun Ren, Wei Han, Guoqiang Jing, Le Gao, Meizhen Bao, Hong Guan

**Affiliations:** Department of Cardiology, Inner Mongolia Medical University Affiliated Hospital, Hohhot, Inner Mongolia, China

**Keywords:** distal radial artery access, acute coronary syndrome, percutaneous coronary intervention, radial artery occlusion, transradial access

## Abstract

**Objective:**

The objective of this study was to evaluate the efficacy and safety of distal transradial access (dTRA) for non-emergent percutaneous coronary intervention (PCI) in patients with acute coronary syndrome (ACS).

**Methods:**

This single-center, randomized controlled study was conducted in the Department of Cardiology at Inner Mongolia Medical University Affiliated Hospital between January and July 2025. Patients with ACS who met the inclusion criteria were assigned to the distal transradial access (dTRA) group or the conventional transradial access (TRA) group at a 1:1 ratio using a random number table method. Clinical baseline data and operation data were collected. Radial artery Doppler ultrasound was performed 24 h and 30 days after PCI to detect radial artery occlusion (RAO).

**Results:**

A total of 106 patients were included in the study, with no significant differences in clinical baseline data and operation data between the two groups. The incidence of RAO at 24 h after PCI in the dTRA group was significantly lower than that in the TRA group (1.9% vs. 15.1%, *P* = 0.037). However, this big difference was not significant at 30 days (0% vs. 5.7%, *P* = 0.241). The TRA group had a longer median compression hemostasis time (274 min vs. 200 min, *P* < 0.001). The median number of punctures, puncture time, and contrast agent dosage between the two groups were similar. The crossover rate in the dTRA and TRA groups was 7.5% and 5.7%, respectively. The incidences of forearm pain, bleeding, and swelling at the puncture site during and after PCI between the two groups were also similar. Logistic regression analysis identified dTRA (OR: 0.075; 95% CI: 0.008–0.723; *P* = 0.025) as an independent protective factor against RAO at 24 h, reducing the risk of the outcome by 92.5%.

**Conclusions:**

For ACS patients undergoing non-emergent PCI, dTRA is a safe and effective approach. dTRA significantly reduced the incidence of RAO at 24 h after PCI and had a shorter hemostasis time, without affecting the interventional procedure.

## 1 Introduction

Percutaneous coronary intervention (PCI) is an important treatment for coronary heart disease. Among the commonly used access routes for PCI, radial artery access (TRA) and femoral artery access are the most prevalent. TRA has become the clinical first choice due to its advantages, including reduced bleeding events, reduced need for blood transfusion, fewer vascular-related complications, improved patient comfort and compliance, lower mortality rate of patients with acute coronary syndrome (ACS), shorter hospital stays, and reduced rates of net adverse clinical events (NACE)—a composite of death, myocardial infarction, stroke, major bleeding, and vascular complications—at 30 days (1–5). At present, over 90% of PCI procedures in China are performed via TRA (6, 7). However, radial artery occlusion (RAO) is one of the common complications after PCI via TRA, with its incidence varying greatly across different studies, ranging from 1% to 33% (8). The occurrence of RAO limits the possibility of repeated PCI via the radial artery and restricts the choice of conduit for coronary artery bypass grafting (CABG) (9). Since Kiemeneij (10) first reported the clinical study of PCI via the left anatomical snuffbox distal transradial artery access (dTRA) in 2017, dTRA has gained increasing attention in various cardiac centers. Subsequent clinical observations have demonstrated that dTRA offers several advantages, such as better patient experience, shorter postprocedural compression time, and a significantly reduced incidence of RAO (10–13). Compared with traditional TRA, dTRA is considered to offer multiple advantages, but there is limited data on the therapeutic effect of non-emergent PCI via dTRA in ACS patients. Therefore, this study aimed to explore the differences in therapeutic effects between dTRA and traditional TRA in ACS patients undergoing non-emergent PCI.

## 2 Materials and methods

### 2.1 Study design and participants

This was a single-center, randomized controlled study conducted in the Department of Cardiology at Inner Mongolia Medical University Affiliated Hospital between January and July 2025. Patients diagnosed with ACS who required non-emergent PCI were enrolled and divided into the TRA group (control group) or the dTRA group (experimental group) using a random number table method. An independent statistician generated random numbers (00–99) for patients numbered 1–106 (by enrollment order), using the Random Number Table for Common Use in Medical Statistics (Appendix of Medical Statistics 4th Edition, edited by Sun Zhenqiu), starting from Row 8, Column 5. Patients with odd random numbers were assigned to the TRA group, while those with even numbers were assigned to the dTRA group. A 1:1 allocation ratio was preset, with 53 patients in each group (no obvious imbalance). Grouping results were stored in sealed envelopes with corresponding numbers, which were opened to confirm the final grouping within 24 h prior to the patient's surgery. Inclusion criteria were as follows: (1) age 18–80 years, regardless of gender; (2) height ≤180 cm (14); (3) no contraindications to anticoagulant and antiplatelet therapy; (4) normal radial artery pulse on preoperative palpation; and (5) provision of signed informed consent. Exclusion criteria were as follows: (1) radial artery occlusion, absence, or severe tortuosity (the radial artery showing ≥2 consecutive tortuosities, each tortuosity angle >90°, under ultrasound, or a single tortuosity angle >120°, with the tortuosity site <5 cm from the puncture point); (2) previous radial artery arteriovenous fistula formation; (3) previous history of angiography or intervention via TRA; (4) undergoing emergency PCI or receiving thrombolytic therapy; (5) patients with cardiogenic shock, severe liver and kidney dysfunction, coagulation disorders, or malignant tumors; (6) patients with previous CABG; and (7) patients who refused to participate in the study. Both the protocol and study were approved by the Ethics Committee of the Inner Mongolia Medical University Affiliated Hospital (YJ2025013), and all patients signed informed consent before enrollment.

### 2.2 Study procedure

All patients received routine preoperative medication (a loading dose of 300 mg aspirin and 600 mg clopidogrel/180 mg ticagrelor + low-molecular-weight heparin anticoagulation for patients with myocardial infarction) and underwent the Allen test. All patients underwent radial artery ultrasound examination preoperatively to confirm the absence of RAO, or severe tortuosity, and to measure the diameter of the radial artery at the puncture site. After disinfection and anesthesia, the Seldinger technique was used to perform the puncture. In the TRA group, puncture was performed at the point with the strongest radial artery pulse approximately 2 cm above the right radial styloid process. In the dTRA group, puncture was performed at the point with the strongest distal radial artery pulse in the right anatomical snuffbox area. Following successful puncture, a 16-cm-long 6F arterial sheath (Terumo Corporation, Japan) with an outer diameter of 2.62 mm was inserted along guidewire. For coronary arteriography, 3,000 U heparin was routinely administered for anticoagulation, and for patients who need coronary intervention procedures, weight-based anticoagulation (heparin dosing 100 U/kg) was adopted. The formulation of intraoperative interventional treatment plans were decided by the operators according to the clinical condition of each patient. After the procedure, the arterial sheath was removed. Patients in the TRA group used an air power compression device (Jilin Ruixiang Kangtai Trading Co., Ltd.) for pressure hemostasis for 4–6 h, while patients in the dTRA group used elastic bandages for pressure hemostasis for 2–4 h. The procedure was performed by two experienced operators. RAO was detected by Doppler ultrasound 24 h and 30 days after the operation within a 5-cm range around the puncture site, evaluated by an independent researcher not involved in the intervention.

### 2.3 Study endpoints

The primary study endpoint was radial artery puncture-related complications, assessed by Doppler ultrasound at 24 h and 30 days after PCI. These included the incidence of RAO, arteriovenous fistula, and pseudoaneurysms. Secondary endpoints included the following: (1) crossover rate, puncture number and time, contrast agent dosage, and compression hemostasis time; (2) forearm pain during and after the operation, puncture site hematoma that was defined using the modified Early Discharge After Transradial Stenting of Coronary Arteries (mEASY) study criteria (15), and palpation of radial artery pulse during follow-up at 24 h and 30 days after PCI; and (3) major adverse cardiac and cerebral events (MACCE) within 30 days postoperatively, defined as a composite endpoint of “cardiovascular death, non-fatal myocardial infarction, revascularization, and stroke.”

### 2.4 Statistical methods

This study adopted both intention-to-treat (ITT) and as-treated (AT) analysis sets. The ITT set, designated as the primary analysis set, included all 106 patients, regardless of whether they completed follow-up or had crossovers. The AT analysis excluded patients who had experienced crossovers and were lost to follow-up (48 in TRA group and 46 in dTRA group). The primary endpoint was analyzed using both ITT and AT analyses. The secondary endpoints were analyzed using only the ITT approach.

The Holm–Bonferroni method was utilized to perform multiplicity adjustment. Secondary endpoints were ranked by their original *p*-values in ascending order. Sequential testing was performed using adjusted *α* levels. If the *p*-value of an endpoint was greater than or equal to the adjusted *α*, all subsequent endpoints were deemed statistically non-significant.

All indicators were analyzed using SPSS 23.0 statistical software (Chicago, IL, USA) and tested for normal distribution. Measurement data conforming to normal distribution were expressed as mean ± SD, and *t*-test was used for comparison between groups. Measurement data with non-normal distribution were expressed as median (interquartile range), and Wilcoxon rank-sum test was used for comparison between groups. Counting data were expressed as *n* (%), and Chi-square test was used for comparison between groups. Bilateral *P* < 0.05 indicated a statistically significant difference. The logistic regression model for analyzing “independent factors of RAO” in this study included the core independent variable “dTRA” and the following 11 covariates: sex, age, hypertension, diabetes mellitus, peripheral vascular disease, smoking history, stent implantation, LVEF, sheath-to-artery diameter ratio, number of puncture attempts, and hemostasis time (in Supplementary Table S1).

## 3 Results

### 3.1 Baseline characteristics

**Figure 1:**
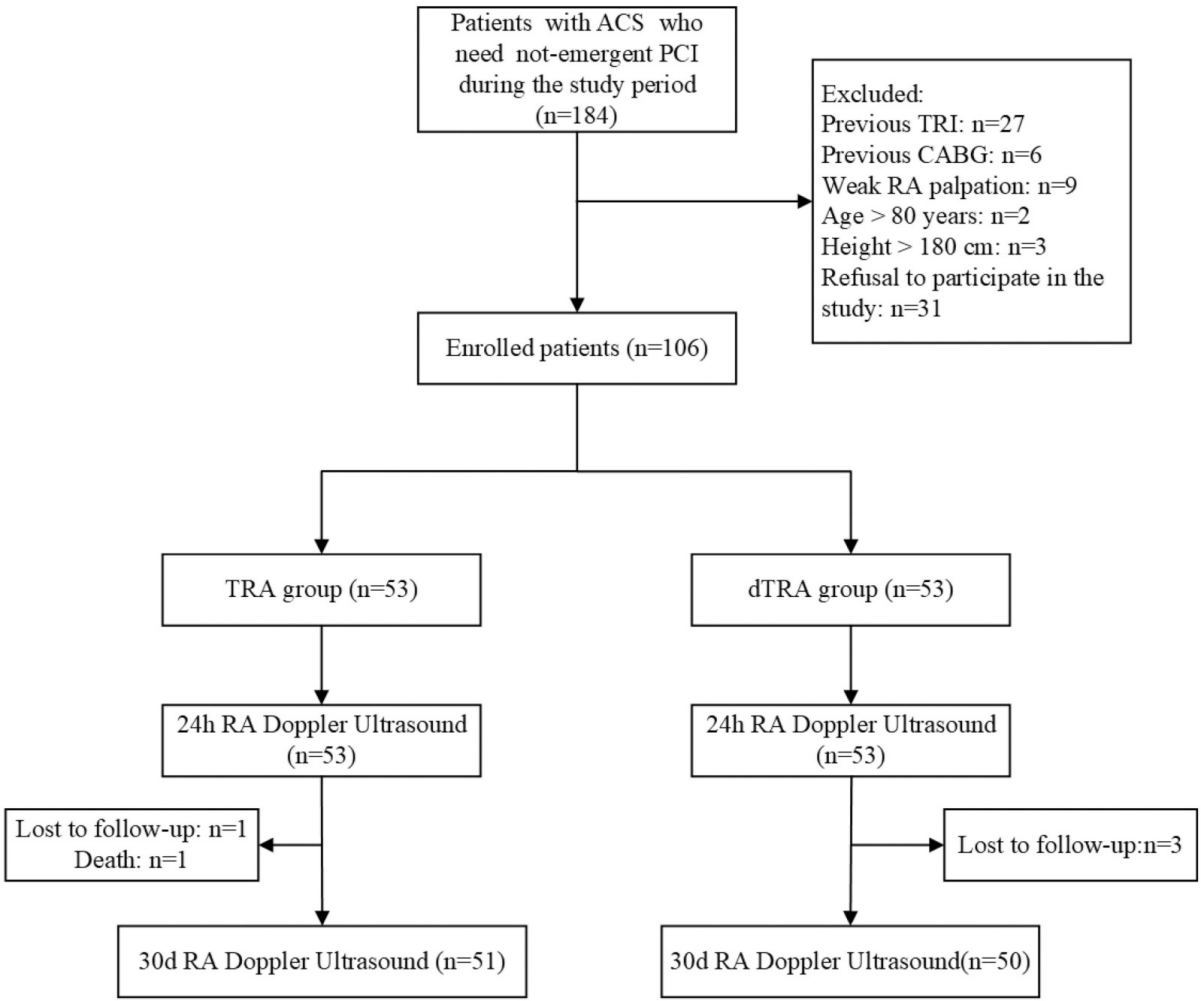
The flow chart for recruitment. ACS, acute coronary syndrome; CABG, coronary artery bypass grafting; dTRA, distal transradial access; PCI, percutaneous coronary intervention; RA, radial artery; TRA, transradial access; TRI, transradial intervention.

During the study period, a total of 106 patients with ACS who met the inclusion criteria were enrolled and assigned to the TRA group or dTRA group in a 1: 1 ratio. Details of recruitment are presented in Figure 1. The clinical baseline data of patients in the TRA group and dTRA group, such as gender composition, age, body mass index, main laboratory indicators, risk factors or underlying disease history, medication use, types of ACS clinical diagnosis, and postoperative hospital stay, showed no significant differences. The comparison of clinical baseline data between the two groups is provided in Table 1.

**Table 1:** Baseline characteristics between two groups.

Variables	Total (*n* = 106)	TRA group (*n* = 53)	dTRA group (*n* = 53)	*P*-value
Gender (male)	86 (81.1)	43 (81.1)	43 (81.1)	1.000
Age (years)	60.3 ± 11.2	60.8 ± 11.8	59.5 ± 10.6	0.512
Height (cm)	169.0 ± 6.5	168.9 ± 6.5	169.4 ± 6.3	0.659
Weight (kg)	72.3 ± 12.1	72.3 ± 11.6	72.3 ± 12.5	0.994
BMI (kg/m^2^)	25.2 ± 3.4	25.3 ± 3.4	25.1 ± 3.5	0.784
Laboratory examinations				
LVEF (%)	65.6 (61.8, 68.8)	66.0 (62.8, 69.0)	65.0 (60.0, 68.0)	0.326
APTT (s)	34.7 ± 3.9	34.8 ± 4.9	34.7 ± 3.4	0.901
Hemoglobin (g/L)	139.7 ± 14.1	139.3 ± 13.8	140.1 ± 14.3	0.309
Platelets (10^9^ /L)	231.3 ± 62.3	234.1 ± 68.4	228.6 ± 58.5	0.408
Medical history				
Smoking history	44 (41.5)	21 (39.6)	23 (43.4)	0.693
Hypertension	60 (56.6)	33 (62.3)	27 (50.9)	0.240
Diabetes mellitus	34 (32.1)	15 (28.3)	19 (35.8)	0.405
Hyperlipidemia	82 (77.4)	39 (73.6)	43 (77.4)	0.353
Cerebrovascular disease	12 (11.3)	6 (11.3)	6 (11.3)	1.000
Atrial fibrillation	3 (2.8)	1 (1.9)	2 (3.8)	0.558
Peripheral vascular disease	6 (5.7)	1 (1.9)	5 (9.6)	0.093
Renal insufficiency	0 (0)	0 (0)	0 (0)	–
Family history	0 (0)	0 (0)	0 (0)	–
Previous MI	8 (7.5)	3 (5.7)	5 (9.4)	0.462
Previous PCI	8 (7.5)	2 (3.8)	6 (11.3)	0.141
Medication history				
Aspirin	103 (97.2)	51 (96.2)	52 (98.1)	0.558
Clopidogrel	98 (92.5)	47 (88.7)	51 (96.2)	0.141
Ticagrelor	7 (6.6)	4 (7.5)	3 (5.7)	0.696
Nitrates	101 (95.3)	50 (94.3)	51 (96.2)	0.647
CCBs	18 (17.0)	9 (17.0)	9 (17.0)	1.000
Statins	104 (98.1)	51 (96.2)	53 (100.0)	0.153
Diagnosis				0.767
STE-ACS	13 (12.3)	7 (13.2)	6 (11.3)	
NSTE-ACS	93 (87.7)	46 (86.8)	47 (87.7)	
Postoperative hospital stay (days)	2.0(1.0, 3.8)	2.0(1.0, 4.0)	2.0(1.0, 3.3)	0.869

Continuous variables presented as mean ± standard deviation or median (Q1, Q3). Categorical variables presented as number (percentage). APTT, activated partial thromboplastin time; BMI, body mass index; CABG, coronary artery bypass grafting; CCB, calcium channel blockers; dTRA, distal transradial access; LVEF, left ventricular ejection fraction; MI, myocardial infarction; NSTE-ACS, non-ST-segment elevation acute coronary syndrome; PCI, percutaneous coronary intervention; STE-ACS, ST-segment elevation acute coronary syndrome; TRA, transradial access.

### 3.2 Operation data

The median duration of angiography and intervention procedure was approximately the same in both groups. There were no significant differences in the number of diseased vessels, the proportion of patients with left main disease, coronary chronic total occlusion, in-stent restenosis, or the culprit vessels. In terms of intervention types, 37.7% of patients in the TRA group underwent coronary arteriography (CAG), 15.1% underwent percutaneous transluminal coronary angioplasty (PTCA), and 47.2% underwent stent implantation. The corresponding indicators in the dTRA group were 28.8%, 17.0%, and 54.7%, respectively, with no significant differences. The comparison of operation data between the two groups is presented in Table 2.

**Table 2:** Procedural characteristics between two groups.

Variables	Total (*n* = 106)	TRA group (*n* = 53)	dTRA group (*n* = 53)	*P*-value
Angiography duration (min)	5.0 (4.6, 6.5)	5.0 (4.0, 6.0)	5.0 (5.0, 7.0)	0.393
Interventional procedure duration (min)	26.0 (0.0, 42.5)	26.0 (0.0, 43.8)	26.0 (0.0, 42.0)	0.974
RA diameter at puncture site	2.5 (2.1, 2.6)	2.7 (2.3, 2.8)	2.6 (2.3, 2.6)	0.154
Sheath-to-artery ratio	1.0 (0.9, 1.1)	1.0 (0.9, 1.1)	1.0 (0.9, 1.0)	0.156
Number of diseased vessels (vessels)				0.269
0	10 (9.4)	7 (13.2)	3 (5.7)	
1	19 (17.9)	9 (17.0)	10 (18.9)	
2	31 (29.2)	11 (20.8)	20 (37.7)	
3	46 (43.4)	26 (49.1)	20 (37.7)	
LMCA	12 (11.3)	7 (13.2)	5 (9.4)	0.540
CTO	15 (14.2)	6 (11.3)	9 (17.0)	0.403
In-stent restenosis	2 (1.9)	1 (1.9)	1 (1.9)	1.000
Target vessel				0.900
None	32 (30.2)	16 (30.2)	16 (30.2)	
LAD	33 (31.1)	18 (34.0)	15 (28.3)	
LCX	12 (11.3)	6 (11.3)	6 (11.3)	
RCA	39 (36.8)	13 (24.5)	16 (30.2)	
Type of intervention				0.627
CAG	35 (33.0)	20 (37.7)	15 (28.8)	
PTCA	17 (16.0)	8 (15.1)	9 (17.0)	
Stent implantation	54(50.9)	25(47.2)	29(54.7)	

Continuous variables presented as median (Q1, Q3). Categorical variables presented as number (percentage). CAG, coronary angiography; CTO, chronic total occlusion; dTRA, distal transradial access; LAD, left anterior descending artery; LCX, left circumflex artery; LMCA, left main coronary artery; PTCA, percutaneous transluminal coronary angioplasty; RA, radial artery; RCA, right coronary artery; TRA, transradial access.

### 3.3 Primary endpoints

All patients underwent radial artery Doppler ultrasound analysis 24 h after PCI. In the ITT analysis, the incidences of RAO, arteriovenous fistula, and pseudoaneurysm at 24 h in the TRA group were 15.1%, 0%, and 1.9%, respectively; in the dTRA group, these were 1.9%, 0%, and 0%, respectively. The difference in RAO incidence between the two groups was statistically significant (*P* = 0.037). In the AT analysis, RAO occurred in 16.7% and 2.2% of patients in the TRA and dTRA groups, respectively (*P* = 0.042). Logistic regression analysis identified dTRA (OR: 0.075; 95% CI: 0.008–0.723; *P* = 0.025) as an independent protective factor against RAO at 24 h, reducing the risk of the outcome by 92.5%.

**Figure 2:**
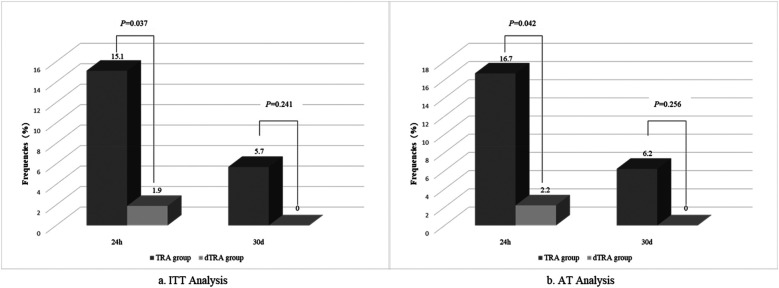
Comparison of RAO incidence analyzed by Doppler ultrasound at 24 h and 30 days between the two groups by ITT and AT analysis. AT, as-treated; dTRA, distal transradial access; ITT, Intention-to-Treat; RAO, radial artery occlusion; TRA, transradial access.

At the 30-day follow-up, two out of 53 patients in the TRA group and three out of 53 patients in the dTRA group failed to complete the Duplex examination. This was because one patient in the TRA group died in a car accident, while the other patient in the TRA group and all three patients in the dTRA group were from other cities and were unable to return due to transportation issues. Three patients in the TRA group continued to have RAO, with no arteriovenous fistula or pseudoaneurysm found. In the dTRA group, no obvious vascular complications such as RAO, pseudoaneurysm, or arteriovenous fistula were observed. ITT (5.7% vs. 0%, *P* = 0.241) and AT (6.2% vs. 0%, *P* = 0.256) analyses showed no difference between two groups. A comparison of RAO incidence between the two groups at 24 h and 30 days after PCI in both ITT and AT analyses is presented in Figure 2.

### 3.4 Secondary endpoints

There were no significant differences in the median number of punctures, puncture time, or contrast agent dosage between the two groups. Patients in the TRA group had a longer compression hemostasis time (274 min vs. 200 min, *P* < 0.001). The overall incidence of crossover in all the patients was 6.6%; although the dTRA group had a higher crossover rate, the difference was not statistically significant (5.7% vs. 7.5%, *P* = 0.696). In the TRA group, three patients crossed over: two crossed over to the contralateral radial artery and one crossed over to the femoral artery. In the dTRA group, four patients crossed over: two crossed over to ipsilateral TRA, one crossed over to the femoral artery, and one crossed over to the brachial artery.

There were no significant differences in the incidences of intraoperative and postoperative forearm pain between the two groups. Three cases were recorded with puncture site hematoma and all were mEASY Ib (3.8% vs. 1.9%, *P* = 0.558). In the TRA group, seven patients (13.2%) had no palpable radial artery pulse at 24 h; in the dTRA group, only one patient (1.9%) had no palpable radial artery pulse, with a statistically significant difference (*P* = 0.031). At the 30-day outpatient follow-up, radial artery palpation assessment was conducted again. In the TRA group, three patients (5.7%) still had no palpable radial artery pulse; in the dTRA group, nobody had absent radial artery pulse, with a statistically significant difference (*P* = 0.046). After adjustment, all the secondary endpoints mentioned earlier showed no significant differences (adjusted *P* > 0.05). There was no statistical difference in MACCE incidence between the two groups (*P* = 0.475). No cardiovascular death or stroke occurred in either group. The TRA group had one case of non-fatal myocardial infarction (1.9%) and one case of target vessel revascularization (1.9%), while the dTRA group had no cases of MACE events (0%). The secondary endpoint comparison between the two groups is presented in Table 3.

**Table 3:** Secondary endpoints between two groups.

Variables	Total (*n* = 106)	TRA group (*n* = 53)	dTRA group (*n* = 53)	*P*-value
Number of punctures	1.0 (1.0, 1.0)	1.0 (1.0, 1.0)	1.0 (1.0, 1.0)	0.168
Puncture duration (min)	3.0 (2.0, 4.0)	3.0 (2.0, 4.0)	3.0 (2.0, 4.0)	0.870
Crossover	7 (6.6)	3 (5.7)	4 (7.5)	0.696
Contrast agent dosage (mL)	160.0 (74.0, 180.0)	160.0 (70.0, 180.0)	160.0 (80.0, 180.0)	0.278
Compression hemostasis duration (min)	241.0 (215.0, 278.0)	274.0 (245.0, 307.0)	200.0 (189.0, 210.0)	0.000
Intraoperative forearm pain	4 (3.8)	3 (5.7)	1 (1.9)	0.308
Postoperative forearm pain	5 (4.7)	3 (5.7)	2 (3.8)	0.647
Puncture site hematoma	3 (2.8)	2 (3.8)	1 (1.9)	0.558
Postoperative 24 h pulse palpation				0.031
Present	98 (92.5)	46 (86.8)	52 (98.1)	
Absent	8 (7.5)	7 (13.2)	1 (1.9)	
Postoperative 30-day pulse palpation				0.046
Present	103 (97.2)	50 (94.3)	53 (100)	
Absent	3 (2.8)	3 (5.7)	0 (0)	
MACCE	2(1.9)	2(3.8)	0(0)	0.475

Continuous variables presented as median (Q1, Q3). Categorical variables presented as number (percentage). dTRA, distal transradial access; MACCE, major adverse cardiac and cerebral events; TRA, transradial access.

## 4 Discussion

This study compared the effectiveness and safety of dTRA and TRA in patients with ACS undergoing non-emergent PCI. The main results showed that compared with TRA, dTRA significantly reduced the incidence of RAO 24 h after PCI and had a shorter hemostasis time, without increasing the number of punctures, puncture and intervention time, or contrast agent dosage. It also did not affect the formulation of interventional strategies.

Although RAO typically has no obvious symptoms, it limits the use of the radial artery as a puncture site in future interventional procedures, as a donor vessel for CABG, and as a site for creating arteriovenous fistulas in patients who need dialysis. The reported incidence of RAO in the “real world” varies widely (1%–33%) (8). At present, multiple strategies for preventing RAO have been reported, such as “patent hemostasis,” higher doses of heparin, and shorter hemostasis times (16). Our study indicates that performing non-emergent PCI via the dTRA approach is a safe and effective alternative complementary strategy that can further reduce the incidence of RAO.

A single-center randomized controlled study (15) involving 1,042 patients randomly assigned patients to the dTRA group and TRA group in a 1:1 ratio. The results showed that the incidence of RAO in the dTRA group was significantly lower than that in the TRA group (3.7% vs. 7.9%, *P* = 0.014). The RAPID III study (17) showed that emergency PCI via dTRA in patients with acute ST-segment elevation myocardial infarction could also reduce the incidence of RAO (1.9% vs. 8.5%, *P* = 0.001). The higher incidence of RAO in the TRA group of the present study compared with that reported in the RAPID III trial (17) (8.7%) can be explained as follows: A key procedural difference lies in the intra-sheath administration of a “cocktail” comprising 200 μg nitroglycerin and 2.5 mg verapamil in the RAPID III trial. This pharmacologic intervention exerts a vasodilatory effect on the radial artery, which not only alleviates mechanical friction between the sheath/catheter and the arterial wall but also mitigates iatrogenic radial artery trauma. Collectively, these effects contribute to the lower RAO incidence observed in the RAPID III trial. In addition, the intraoperative anticoagulation level is also one of the factors that may affect RAO rate. Previous studies have shown that a heparin dose of >50 IU/kg reduces the RAO risk by 80% (18). The RAPID III study included only patients with ST-segment elevation myocardial infarction (STEMI) and used 100 IU/kg unfractionated heparin intraoperatively. In our study, which included ACS patients, 3,000 IU of heparin was administered during the initial coronary angiography; if further interventional treatment was required, an additional 100 IU/kg of heparin was supplemented. Thus, the anticoagulation level in our study was slightly lower than that in the RAPID III study. Notably, the relatively small sample size of the present study may have introduced potential bias into the observed results, which could partially account for the discrepancy in RAO rates between the two studies. Another prospective randomized controlled study (19) used Doppler ultrasound to examine the proximal radial artery for RAO at 24 h and 30 days after PCI. The results demonstrated that the incidences of RAO at 24 h and 30 days in the TRA group were 8.4% and 5.6%, respectively, while those in the dTRA group were 0.7% and 0.7%, respectively. These rates were significantly lower in the dTRA group than those in the TRA group (*P* < 0.05). This approach reduced the absolute risk of RAO by 7.7% at 24 h after surgery and by 4.9% and at 30 days. The results of this study showed that compared with TRA, the incidence of RAO at 24 h in the dTRA group was significantly reduced (15.9% vs. 1.9%, *P* = 0.037). At 30 days, the incidences of RAO were 5.7% vs. 0% (*P* = 0.248), which were generally consistent with the above-mentioned research results. The most likely pathophysiological explanation for the reduced incidence of RAO with dTRA is the rich anastomotic network between the deep and superficial arterial branches in the wrist and hand area, which helps maintain blood flow in the forearm radial artery and thus contributes to reducing the incidence of RAO.

Shortening the hemostasis time and using patent hemostasis techniques are two key factors in reducing the incidence of RAO. Different techniques can change both the pressure and time of compression (16). The characteristics of postoperative compression hemostasis differ according to access routes, and their impacts on the occlusion incidence also vary. The puncture site of TRA is relatively deep. During postoperative compression hemostasis, greater pressure and longer time are required to ensure the hemostasis effect, which increases the risk of radial artery blood flow obstruction to a certain extent, thus raising the incidence of RAO. In contrast, the puncture site of dTRA is superficial, and hemostasis is relatively easy, requiring less compression pressure and time. Some studies have compared the postoperative compression hemostasis of the two access routes and found that the average compression time after dTRA is significantly shorter than that after TRA and the incidence of RAO is lower (15, 17). These findings indicate that reasonable compression hemostasis methods and time are crucial for reducing the incidence of RAO, and dTRA has certain advantages in this regard. The PROPHET Ⅱ study, based on the technique of promoting patent hemostasis of the radial artery through brief ulnar artery compression, evaluated the patent hemostasis technique using ulnar artery compression. The incidences of RAO at 24 h and 30 days after PCI measured by plethysmography were 1.0% and 0.9%, respectively (20, 21). A large-scale meta-analysis reported an early incidence of RAO by plethysmography of 7.7%, increasing to 10% when using ultrasound examination (8), considering that Doppler ultrasound is more sensitive in detecting postoperative RAO. Moreover, laser perfusion imaging (LPI), as described by Indolfi et al., has been a reliable, quick, and easy diagnostic technique for RAO. As a novel non-invasive approach, laser Doppler scanning enables operator-independent, rapid RAO diagnosis following catheterization via a low-power laser beam that generates blood perfusion color maps. It outperforms traditional methods by measuring whole-hand blood flow (avoiding site variability bias), being faster than vascular Doppler (the reference standard), and featuring ease of use plus non-contact operation (preventing artifacts) (22, 23). The CRASOC Ⅲ study used a thinner introducer sheath and confirmed the correlation between hemostasis time and RAO. The study found that when compressed with a 10-mL inflation pressure for 90 min, the incidence of RAO measured by ultrasound was the lowest (0.6%) (24). Moreover, performing radial artery puncture under ultrasound guidance and following a standardized operation process may further reduce the incidence of RAO (15).

Most previous studies focused on emergency STEMI or strictly selected populations with few comorbidities. In contrast, this study was conducted in a routine general hospital and validated the safety and superiority of dTRA in a broad-spectrum non-emergent ACS population without strict exclusion of mild-to-moderate comorbidities. The results demonstrated significantly lower RAO incidence in the dTRA group than in the TRA group with no difference in complications, consolidating evidence for the application of dTRA in ordinary clinical patients and addressing the practical question of “whether conclusions from strictly selected populations can be directly applied.”

However, dTRA puncture also has some disadvantages. The lumen diameter is relatively smaller, the blood vessel has a more tortuous course, the puncture is more difficult, the technical requirements are higher, and the learning curve is steeper. These aspects may prolong the operation time and increase the risk of puncture failure (15), but they offer significant long-term safety and clinical benefits. This study demonstrated no differences in the puncture success rate, crossover rate, and operation duration. Considering the continuous learning and application of dTRA in various cardiac centers, specialists at our center can now proficiently perform coronary angiography and intervention through dTRA. However, the sample size of this study was small, and the proportion of patients with complex lesions was low, which may have an impact on the result analysis. In addition, the smaller lumen of the distal radial artery also limits the application of transradial artery in complex coronary lesions. However, the use of thin-walled sheaths in clinical practice makes it possible to complete interventional treatments for left main bifurcation lesions, chronic occlusive lesions, rotational atherectomy, and reverse angulated bifurcation lesions via dTRA (25).

This study has certain limitations. It was carried out at a single center with a small sample size. During the design phase, the core goal was to “summarize real clinical data,” so we failed to complete the *a priori* sample size calculation in accordance with the standards of prospective trials. Future studies will strictly standardize this process. This study used the odd–even allocation method based on a random number table instead of a centralized computer-generated random sequence, which may have affected the robustness of allocation concealment and could carry the risk of potential selection bias. Although there was no obvious imbalance in baseline data and confounding was reduced through statistical adjustment, the possibility of selection bias cannot be completely excluded. In the future, multicenter, large-sample, long-term randomized controlled studies are warranted. Such studies should adopt centralized computer-generated random sequences to improve methodological rigor, use ultrasound-guided puncture to ensure procedural standardization, and flatten learning-curve effects to further explore the potential value of performing non-emergent PCI via dTRA and reduce the occurrence of RAO.

## 5 Conclusion

In conclusion, non-emergent PCI via dTRA in ACS patients is safe and effective, which can reduce the occurrence of RAO and shorten hemostasis time without increasing the puncture and intervention time.

## Data Availability

The original contributions presented in the study are included in the article/Supplementary Material; further inquiries can be directed to the corresponding author.

## References

[1] Jolly SS Amlani S Hamon M Yusuf S Mehta SR. Radial versus femoral access for coronary angiography or intervention and the impact on major bleeding and ischemic events: a systematic review and meta-analysis of randomized trials. Am Heart J. (2009) 157(1):132–40. 10.1016/j.ahj.2008.08.023 19081409

[2] Jolly SS Yusuf S Cairns J Niemelä K Xavier D Widimsky P Radial versus femoral access for coronary angiography and intervention in patients with acute coronary syndromes (RIVAL): a randomised, parallel group, multicentre trial. Lancet. (2011) 377(9775):1409–20. 10.1016/s0140-6736(11)60404-2 21470671

[3] Valgimigli M Gagnor A Calabró P Frigoli E Leonardi S Zaro T Radial versus femoral access in patients with acute coronary syndromes undergoing invasive management: a randomised multicentre trial. Lancet. (2015) 385(9986):2465–76. 10.1016/s0140-6736(15)60292-6 25791214

[4] Vranckx P Frigoli E Rothenbühler M Tomassini F Garducci S Andò G Radial versus femoral access in patients with acute coronary syndromes with or without ST-segment elevation. Eur Heart J. (2017) 38(14):1069–80. 10.1093/eurheartj/ehx048 28329389

[5] Scalise RFM Salito AM Polimeni A Ruiz VG Virga V Frigione P Radial artery access for percutaneous cardiovascular interventions: contemporary insights and novel approaches. J Clin Med. (2019) 8(10):1–19. 10.3390/jcm8101727 PMC6833028 31635342

[6] Lu Y Zhang H Wang Y Zhou T Welsh J Liu J Percutaneous coronary intervention in patients without acute myocardial infarction in China: results from the China PEACE prospective study of percutaneous coronary intervention. JAMA Netw Open. (2018) 1(8):e185446. 10.1001/jamanetworkopen.2018.5446 30646292 PMC6324328

[7] Zhao R Xu K Li Y Qiu M Han Y, NRCIMH Program. Percutaneous coronary intervention in patients with acute coronary syndrome in Chinese Military Hospitals, 2011–2014: a retrospective observational study of a national registry. BMJ Open. (2018) 8(10):e023133. 10.1136/bmjopen-2018-023133 30361405 PMC6224757

[8] Rashid M Kwok CS Pancholy S Chugh S Kedev SA Bernat I Radial artery occlusion after transradial interventions: a systematic review and meta-analysis. J Am Heart Assoc. (2016) 5(1):1–23. 10.1161/jaha.115.002686 PMC4859386 26811162

[9] Mounsey CA Mawhinney JA Werner RS Taggar DP. Does previous transradial catheterization preclude use of the radial artery as a conduit in coronary artery bypass surgery? Circulation. (2016) 134(9):681–8. 10.1161/circulationaha.116.022992 27572880

[10] Kiemeneij F. Left distal transradial access in the anatomical snuffbox for coronary angiography (ldTRA) and interventions (ldTRI). EuroIntervention. (2017) 13(7):851–7. 10.4244/eij-d-17-00079 28506941

[11] Koutouzis M Kontopodis E Tassopoulos A Tsiafoutis I Katsanou K Rigatou A Distal versus traditional radial approach for coronary angiography. Cardiovasc Revasc Med. (2019) 20(8):678–80. 10.1016/j.carrev.2018.09.018 30314833

[12] Soydan E Akın M. Coronary angiography using the left distal radial approach—an alternative site to conventional radial coronary angiography. Anatol J Cardiol. (2018) 19(4):243–8. 10.14744/AnatolJCardiol.2018.59932 29578203 PMC5998856

[13] Valsecchi O Vassileva A Cereda AF Canova P Satogami K Fiocca L Early clinical experience with right and left distal transradial access in the anatomical snuffbox in 52 consecutive patients. J Invasive Cardiol. (2018) 30(6):218–23. 29543187

[14] Expert Panel of Chinese Expert Consensus on Coronary Artery Interventional Diagnosis and Treatment via Distal Radial Artery, Thumb Club. Chinese Expert consensus on coronary artery interventional diagnosis and treatment via distal radial artery. Chin J Interv Cardiol. (2020) 28(12):667–74. 10.3969/j.issn.1004-8812.2020.12.002

[15] Tsigkas G Papageorgiou A Moulias A Kalogeropoulos AP Papageorgopoulou C Apostolos A Distal or traditional transradial access site for coronary procedures: a single-center, randomized study. JACC Cardiovasc Interv. (2022) 15(1):22–32. 10.1016/j.jcin.2021.09.037 34922888

[16] Bernat I Aminian A Pancholy S Mamas M Gaudino M Nolan J Best practices for the prevention of radial artery occlusion after transradial diagnostic angiography and intervention: an international consensus paper. JACC Cardiovasc Interv. (2019) 12(22):2235–46. 10.1016/j.jcin.2019.07.043 31753298

[17] Li Z Wang Y Song J Wang S Wang Y Wu Y Distal radial access to prevent radial artery occlusion for STEMI patients (RAPID III): a randomized controlled trial. BMC Med. (2025) 23(1):173. 10.1186/s12916-025-04005-1 40128873 PMC11934606

[18] Hahalis GN Leopoulou M Tsigkas G Xanthopoulou I Patsilinakos S Patsourakos NG Multicenter randomized evaluation of high versus standard heparin dose on incident radial arterial occlusion after transradial coronary angiography: the SPIRIT OF ARTEMIS study. JACC Cardiovasc Interv. (2018) 11(22):2241–50. 10.1016/j.jcin.2018.08.009 30391389

[19] Eid-Lidt G Rodríguez AR Castellanos JJ Pasos JIF López KEE Gaspar J. Distal radial artery approach to prevent radial artery occlusion trial. JACC Cardiovasc Interv. (2021) 14(4):378–85. 10.1016/j.jcin.2020.10.013 33602433

[20] Koutouzis MJ Maniotis CD Avdikos G Tsoumeleas A Andreou C Kyriakides ZS. ULnar artery transient compression facilitating radial artery patent hemostasis (ULTRA): a novel technique to reduce radial artery occlusion after transradial coronary catheterization. J Invasive Cardiol. (2016) 28(11):451–4. 27529655

[21] Pancholy SB Bernat I Bertrand OF Patel TM. Prevention of radial artery occlusion after transradial catheterization: the PROPHET-II randomized trial. JACC Cardiovasc Interv. (2016) 9(19):1992–9. 10.1016/j.jcin.2016.07.020 27712733

[22] Indolfi C Passafaro F Sorrentino S Spaccarotella C Mongiardo A Torella D Hand laser perfusion imaging to assess radial artery patency: a pilot study. J Clin Med. (2018) 7(10):1–11. 10.3390/jcm7100319 PMC6210442 30279350

[23] Rosa SD Passafaro F Polimeni A Sorrentino S Indolf C. A novel quick and easy test for radial artery occlusion with the laser Doppler scan. JACC Cardiovasc Interv. (2014) 7(8):e89–90. 10.1016/j.jcin.2013.11 25086840

[24] Dangoisse V Guédès A Chenu P Hanet C Albert C Robin V Usefulness of a gentle and short hemostasis using the transradial band device after transradial access for percutaneous coronary angiography and interventions to reduce the radial artery occlusion rate (from the prospective and randomized CRASOC I, II, and III studies). Am J Cardiol. (2017) 120(3):374–9. 10.1016/j.amjcard.2017.04.037 28577752

[25] Zong B Liu Y Han B Feng CG. Safety and feasibility of a 7F thin-walled sheath via distal transradial artery access for complex coronary intervention. Front Cardiovasc Med. (2022) 9:959197. 10.3389/fcvm.2022.959197 36312263 PMC9599392

