# Supplementary material for: The safety and efficacy of non-emergent percutaneous coronary intervention via distal radial artery access in patients with acute coronary syndrome
Source: Front Cardiovasc Med. 2025 Dec 19;12:1696742. doi: 10.3389/fcvm.2025.1696742 (PMC12757397; doi:10.3389/fcvm.2025.1696742)
Supplement: Supplementary file 1 [file Table1.pdf]

Predictors of RAO

The logistic regression model for analyzing "independent factors of RAO" in this study included the core independent variable "dTRA", and the following 11 covariates: sex, age, hypertension, diabetes mellitus, peripheral vascular disease, smoking history, stent implantation, LVEF, sheath-to-artery diameter ratio, number of puncture attempts, and hemostasis time **(Table S1)**.

Table S1. Predictors of RAO in multivariate logistic regression analysis

Variables	OR	95%CI	*P* value
group	0.075	0.008-0.723	0.025
sex	3.280	0.243-44.241	0.371
age	0.974	0.891-1.064	0.560
hypertension	1.379	0.207-9.159	0.740
diabetes mellitus	1.372	0.162-11.601	0.772
peripheral vascular disease	0.943	0.842-1.056	0.082
smoking history	0.452	0.059-3.451	0.444
stent implantation	2.540	0.281-22.920	0.406
LVEF	0.700	0.503-1.975	0.135
sheath-to-artery diameter ratio	0.134	0.502-1.087	0.063
number of puncture attempts	0.784	0.198-3.105	0.729
hemostasis time	1.011	0.985-1.037	0.404
